# Continuous Hepatogonodal and Splenogonogal Fusion: A Rare Cause of Bilateral Intra-Abdominal Testis in an 18-Month-Old Boy

**DOI:** 10.1055/s-0042-1747671

**Published:** 2022-06-25

**Authors:** Gül Durmuş, Ozlem Boybeyi-Turer, Hatice Nursun Özcan, Onur Gözmen, Hüseyin Demirbilek, Tutku Soyer

**Affiliations:** 1 Department of Pediatric Surgery, Hacettepe Universitesi Tip Fakultesi, Ankara, Turkey; 2 Department of Pediatric Radiology, Hacettepe Universitesi Tip Fakultesi, Ankara, Turkey; 3 Department of Pediatric Endocrinology, Hacettepe Universitesi Tip Fakultesi, Ankara, Turkey

**Keywords:** hepatogonadal fusion, splenogonadal fusion, undescended testis

## Abstract

The fusion of gonadal structures with internal organs is very rare. The close proximity between the left gonad and spleen during embryogenesis may result in splenogonadal fusion (SGF). Moreover, the trapping of hepatocyte-destined mesenchyme cells in gonads is defined as hepatogonadal fusion (HGF). The fusion of gonads with intra-abdominal organs may be continuous and may impair testicular descent during the prenatal period. We herein report an 18-month-old boy presented with bilateral nonpalpable testis due to concomitant continuous HGF and SGF. To our knowledge, this is the first case of concomitant HGF and SGF in a boy with bilateral intra-abdominal testis. Laparoscopic excision of fibrous cords and orchidopexy can be achieved despite continuous fusions.

## Introduction

Gonadal structures fused with other intra-abdominal organs are very rare, and adrenal remnant within a paratesticular tissue is the most common type. 1 During organogenesis, close proximity between single cells or aggregates of mesenchymal cells with the gonads may cause splenogonadal fusion (SGF) and/or hepatogonadal fusion (HGF). SGF is more common among these anomalies and first described by Bostroem in 1883. 2 Putschar and Manion classified SGF as continuous and discontinuous types according to the presence of a direct connection between gonad and spleen. 3 The continuous type consists of a cord-like splenic tissue or fibrous tissue embedded with splenic nodules. In the discontinuous type, spleen-destined cells are trapped on paratesticular tissue. 4 HGF is less frequent than SGF, and only five cases have been reported previously. 5 6 7 8 9 Undescended testis is the most common abnormality associated with SGF, with a frequency of 31%. 1 SGF, especially continuous type, may cause undescended testis. In a large series of patients, right and left intra-abdominal testes were reported in 26 and 65% of cases, respectively. 1 Although undescended testis can be bilateral in 59% of patients with continuous SGF, concomitant occurrence of SGF and HGF in the same patient has not been reported before. ^3^ Herein, we report the first case of continuous SGF and HGF as cause of bilateral intra-abdominal testis in children.

## Case Report

**Fig. 1:**
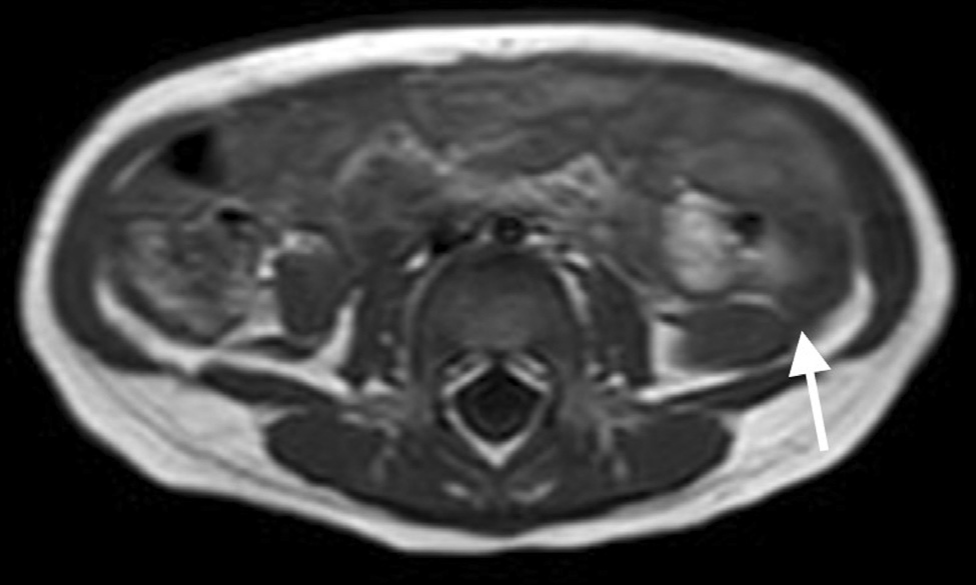
Splenic cord-like structure on magnetic resonance imaging (arrow).

**Fig. 2:**
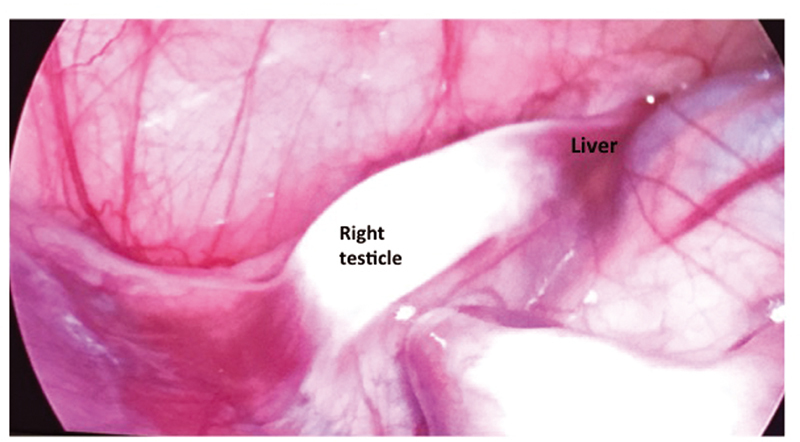
Laparoscopic evaluation revealed hepatogonadal fusion. A short fibrous cord was attached to the right testis and lower aspect of the right liver lobe.

**Fig. 3:**
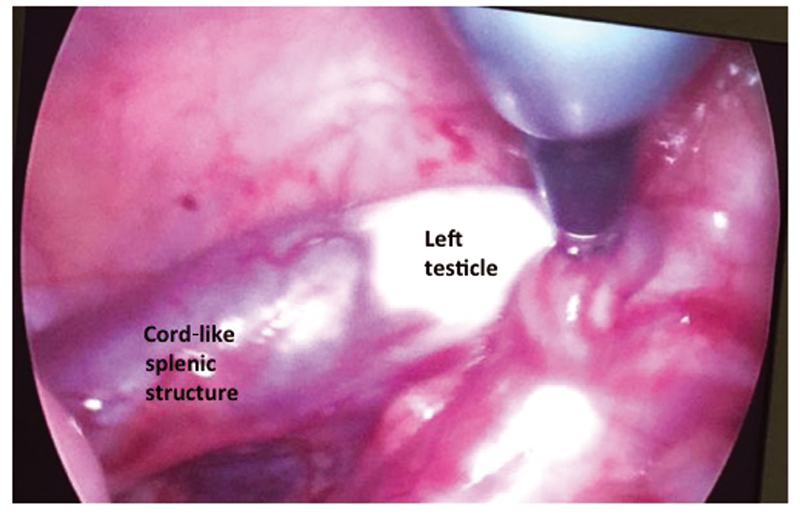
The cord-like structure composed of splenic tissue lies between the spleen and left testicle.

An 18-month-old boy admitted to our department with bilateral undescended testis. In his medical history, he had partial anomalous pulmonary venous return and pulmonary hypertension. At the time of the presentation, his height was 74.5 cm (10–25 p), weight was 8.8 kg (3–10 p), and the head circumference was 45 cm (25–50 p). Other physical findings were unremarkable except for a grade 2 systolic murmur. The genitourinary examination revealed bilateral nonpalpable testis and a stretched penile length of 4.5 cm with no sign of undervirilization. Suprapubic and scrotal ultrasonography did also not show the presence of gonads. Karyotype was normal (46, XY). The gonadotropin, anti-Mullerian hormone, and testosterone levels were also normal. The detectable anti-Mullerian hormone level (23,6 mµ/L) suggested existence of testicular tissue with functional Sertoli cells. A human chorionic gonadotrophin (HCG) stimulation test (3,000 U/kg/day, subcutaneously, for 3 days) was performed. HCG-stimulated testosterone level (562 ng/dL) suggested the presence of functional gonads. The patient underwent abdominal magnetic resonance imaging (MRI) with a presumptive diagnosis of bilateral intra-abdominal testis. Testes were not observed in the scrotum and both groins on ultrasound. Abdominal MRI revealed bilateral intra-abdominal tissue was observed adjacent to the psoas muscles below the kidneys. The fibrous cord like structure between spleen and left testicle was also demonstrated ( Fig. 1 ). After these findings, the patient was consulted to pediatric surgery department. The patient underwent laparoscopic exploration. The right testicle was adherent to the liver with a fibrous band ( Fig. 2 ). The right testicle was 10 × 7 mm in size and the spermatic cord was short with no epididymis anomaly. The vessels of both testicles were apart from the splenic and hepatic tissues. The band was excised using the LigaSure, and the right testicle was replaced to the inguinal canal via the internal ring. On the right side, it was not possible to replace the right testicle in the dartos pouch. Since we can fix the testis, lowermost part of the inguinal canal, any additional procedure such as Stephan's-Fowler or Shehata technique were not considered. The left testicle was also fused to the spleen with a thick fibrous cord of splenic tissue ( Fig. 3 ). The size of left testicle was 12 × 8 mm and it has adequate spermatic cord length. The splenic cord was excised, and the left testicle was easily replaced into a dartos pouch in the scrotum. Histopathology of excised cord confirmed continuous SGF and testicular tissue on the excised splenic cord showed normal histology. Genetics analysis did not find a mutation within the INSL3 gene. Six months after laparoscopy, the patient underwent a second-stage orchidopexy. The right testicle was localized in the inguinal canal and replaced to dartos pouch in the scrotum. Both testicles were of normal in size and location after 2 months follow-up.

## Discussion

Several theories have been suggested in the etiology of SGF and HGF. SGF is thought to occur between the fifth and eighth weeks of gestation. The developing spleen comes into close proximity to the left urogenital fold during rotation of the embryonic gut. This spleen–gonadal relationship remains until the eighth week of development, when gonadal descent is initiated. At this period, the fusion started between the surface of the developing genital ridge and the splenic anlage. 10 Le Roux and Heddle suggest that discontinuous SGF should be categorized as a rare variant of accessory spleen rather than a SGF. 11

SGF occurs predominantly in males with a male-to-female ratio of 16:1, and only nine female cases have been reported. 10 However, the true incidence of female SGF is unclear because of the inaccessibility of the female gonads. Approximately half of the cases are reported to be younger than 10 years old and diagnosed incidentally during a routine groin exploration. The most common presentation of SGF is that of testicular swelling. It has been reported that 37% of cases underwent unnecessary orchiectomy because of suspicion of a testicular tumor. 12 Undescended testis is significantly associated with the continuous type of SGF. 1 The incidence of undescended testis was 44% in continuous and 14% in discontinuous types. 1 The continuous SGF is usually associated with severe congenital anomalies and carries a fivefold increased risk than the discontinuous type. 13 In our case, partial anomalous pulmonary venous return and pulmonary hypertension were accompanied to SGF.

HGF is extremely rare, and only five cases have been reported. 5 6 7 8 9 The mechanism of HGF has been speculated as trapping of hepatocyte-destined mesenchyme cells on gonadal structures. 5 This theory may explain the discontinuous type of HGF similar to other heterotopic liver in other tissues like the umbilical cord, jejunum, and in the thorax. 5 However, it is difficult to define the clear pathogenesis in continuous-type HGF. Fetal testicular peritoneal folds (mesonephric sheath) attaching to the most lateral side of the inferior border of the right liver lobe were reported as a cause of continuous HGF. 8 Different clinical presentations of HGF have been reported. 5 6 7 8 9 Hepatic tissue on gonad can be identified during hernia repair, or fibrous-like pedicle was connected to liver with atrophic or normal gonads (5, 7). Like SGF, HGF may also be associated with undescended testis if fibrous cord-like structure lies between gonad and liver. Four of the patients with HGF present with undescended testes. Herein, we report the sixth pediatric case with HGF. Remarkably, our case showed a continuous type of both SGF and HGF, causing a bilateral undescended testis. To the best of our knowledge, concomitant SGF and HGF as a cause of bilateral intra-abdominal testis have not been reported before.

The treatment of SGF varies according to the type of gonadal fusion. In the past, some authors report unnecessary orchiectomy with a presumptive diagnosis of primary testicular tumor. SGF can be demonstrated by 99Tc sulfur colloid preoperatively. 14 Technetium scans are useful especially in discontinuous type of SGP. Since fibrous-cord like SGF demonstrated in MRI, technetium scans were not useful in this case. As the awareness of SGF increases, the excision of splenic tissue and fibrous cord-like structures becomes the choice of treatment. Laparoscopic exploration of patients with impalpable testis increased the diagnosis of continuous SGF and HGF in children. In addition, a comprehensive preoperative workup for evaluation of the presence of a functional gonad is essential for making the correct diagnosis and most appropriate management option. Indeed, the preoperative hormonal assessment of our case was suggestive for the presence of functional gonads. This has helped us to select the most conservative intervention. Also, the excision of cord-like structures enables orchidopexy in most cases. Orchiectomy should be reserved for patients with atrophic testis. In our patient, fibrous cord-like structures on the right side and splenic cord on the left side were successfully excised by laparoscopy. Although the left testicle was replaced in the dartos pouch, the right testicle could only be replaced at the uppermost level of the right scrotum. Since we could replace the testis in the inguinal channel, we chose a second-stage orchidopexy with inguinal exploration instead of the Shehata procedure. 15 Therefore, the surgical treatment of SGF and HGF depends on the viability of the testicle and the length of the spermatic cord.

In conclusion, concomitant continuous HGF and SGF may cause bilateral undescended testis with intra-abdominal localization. Laparoscopic excision of continuous fibrous cords and orchidopexy can be achieved despite continuous fusions.

## Notes

The New Insight and Importance for the Pediatric Surgeon We present the first case of continuous hepatogonadal and splenogonodal fusion as a cause of bilateral intra-abdominal testis in children. Laparoscopic excision of fibrous cords and orchidopexy can be achieved despite continuous fusions.

## References

[1] Cortes D Thorup J M Visfeldt J The pathogenesis of cryptorchidism and splenogonadal fusion: a new hypothesis Br J Urol 1996 77 02 285 290 8800901 10.1046/j.1464-410x.1996.89022.x

[2] Bostroem E Demonstration of a specimen of fused spleen with left testicle. Society of German natural scientists and physicians negotiations of the 56th assembly Freiburg 1883 14

[3] Putschar W G Manion W C Splenogonadal fusion Am J Pathol 1956 32 01 15 33 13275562 PMC1942585

[4] Werdham H Kumar A Jain N Splenogonodal fusion: case report and review of the literature Pediatr Surg Int 1993 8 58 59

[5] Ferro F Lais A Boldrini R De Peppo F Federici G Bosman C Hepatogonadal fusion J Pediatr Surg 1996 31 03 435 436 8708920 10.1016/s0022-3468(96)90755-1

[6] Lund J M Bouhadiba N Sams V Tsang T Hepato-testicular fusion: an unusual case of undescended testes BJU Int 2001 88 04 439 440 11564037 10.1046/j.1464-410x.2001.02359.x

[7] Fan R Faught P R Sun J Meldrum K K Hepatogonadal fusion J Pediatr Surg 2012 47 02 e5 e6 10.1016/j.jpedsurg.2011.10.071 22325418

[8] El-koutby M ElJalby M Hepatogonadal fusion in cryptorchidism J Pediatr Surg Case Rep 2018 34 4 6

[9] Al-Saied G Ibrahim M Al-Nefaei S Thabet R Mohamed A Hepato-testicular fusion: a rare case of undescended testis J Pediatr Surg Case Rep 2016 4 35 37

[10] Chen S L Kao Y L Sun H S Lin W L Splenogonadal fusion J Formos Med Assoc 2008 107 11 892 895 18971159 10.1016/S0929-6646(08)60206-5

[11] Le Roux P J Heddle R M Splenogonadal fusion: is the accepted classification system accurate? BJU Int 2000 85 01 114 115 10619957 10.1046/j.1464-410x.2000.00410.x

[12] Khairat A BM Ismail A M Splenogonadal fusion: case presentation and literature review J Pediatr Surg 2005 40 08 1357 1360 16080949 10.1016/j.jpedsurg.2005.05.027

[13] Gouw A S Elema J D Bink-Boelkens M T de Jongh H J ten Kate L P The spectrum of splenogonadal fusion. Case report and review of 84 reported cases Eur J Pediatr 1985 144 04 316 323 4076247 10.1007/BF00441771

[14] Steinmetz A P Rappaport A Nikolov G Priel I E Chamovitz D L Dolev E Splenogonadal fusion diagnosed by spleen scintigraphy J Nucl Med 1997 38 07 1153 1155 9225811

[15] Shehata S Shalaby R Ismail M Abouheba M Elrouby A Staged laparoscopic traction-orchiopexy for intraabdominal testis (Shehata technique): stretching the limits for preservation of testicular vasculature J Pediatr Surg 2016 51 02 211 215 26655212 10.1016/j.jpedsurg.2015.10.063

